# Low back pain self-management mobile applications: a systematic review on digital platforms*

**DOI:** 10.1590/1980-220X-REEUSP-2023-0326en

**Published:** 2024-06-14

**Authors:** Zulamar Aguiar Cargnin, Dulcinéia Ghizoni Schneider, Michelle Gonçalves de Souza, Mara Ambrosina de Oliveira Vargas, Francis Solange Vieira Tourinho

**Affiliations:** 1 Universidade Federal de Santa Catarina, Programa de Pós-Graduação em Enfermagem, Florianópolis, SC, Brazil.; 2 Universidade Federal de Santa Catarina, Faculdade de Enfermagem, Programa de Pós-Graduação em Enfermagem, Florianópolis, SC, Brazil.; 3 Secretaria Estadual de Saúde de Santa Catarina, Setor de Fisioterapia, Florianópolis, SC, Brazil.

**Keywords:** Low Back Pain, Mobile Applications, Smartphone, Self-Management, Self Care, Dolor de la Región Lumbar, Aplicaciones Móviles, Teléfono Inteligente, Automanejo, Autocuidado, Dor Lombar, Aplicativos Móveis, Smartphone, Autogestão, Autocuidado

## Abstract

**Objective:**

To identify and analyze the features and quality of self-management support of mobile applications available in Brazil for chronic low back pain in adults.

**Method:**

A systematic review on the Apple Store^®^ and Google Play^®^ digital platforms. The Self-Management Support Assessment Tool scale was used to assess self-management support and the Institute for Healthcare Informatics Functionality Score scale was used to assess functionality.

**Results:**

Seventeen applications were selected, which included around seven self-management skills. The applications that met the majority of self-management support skills were Pathways, Branch, Pancea, Pain Navigator, and Curable. The Curable, Branch and MoovButh applications had the highest scores, with ten features on the functionality scale.

**Conclusion:**

Some applications have the potential to complement in-person treatment in terms of validity, acceptability and clinical usefulness in pain management. However, barriers such as lack of partnership between healthcare providers and patients, limited evidence-based content, social support, cultural relevance, cost, language, security and privacy can limit their sustained use. PROSPERO Registration: CRD42022382686.

## RESUMO

**Objetivo:**

Identificar e analisar os recursos e a qualidade do suporte de autogerenciamento dos aplicativos móveis disponíveis no Brasil para dor lombar crônica em adultos.

**Método:**

Revisão sistemática nas plataformas digitais *Apple Store* ^®^ e *Google Play* ^®^. Utilizaram-se a escala *Self-Management Support Assessment Tool* para avaliar o suporte ao autogerenciamento e a escala *Institute for Healthcare Informatics Functionality Score* para avaliar a funcionalidade.

**Resultados:**

Foram selecionados 17 aplicativos, que incluíram cerca de sete habilidades de autogerenciamento. Os aplicativos que atendiam à maioria das habilidades de suporte ao autogerenciamento foram *Pathways, Branch, Pancea, Pain Navigator* e *Curable*. Os aplicativos *Curable, Branch e MoovButh* apresentaram as maiores pontuações, com dez funcionalidades na escala de funcionalidade.

**Conclusão:**

Alguns aplicativos têm potencial para complementar o tratamento presencial em termos de validade, aceitabilidade e utilidade clínica no manejo da dor. No entanto, barreiras como falta de parceria entre profissionais de saúde e pacientes, conteúdo limitado baseado em evidências, apoio social, relevância cultural, custo, idioma, segurança e privacidade podem limitar o seu uso sustentado. Registro PROSPERO: CRD42022382686.

## RESUMEN

**Objetivo:**

Identificar y analizar las características y la calidad del soporte de autogestión de aplicaciones móviles disponibles en Brasil para el dolor lumbar crónico en adultos.

**Método:**

Revisión sistemática en las plataformas digitales Apple Store*^®^* y Google Play*^®^*. Se utilizó la escala Self-Management Support Assessment Tool para evaluar el apoyo a la autogestión y la escala Institute for Healthcare Informatics Functionality Score para evaluar la funcionalidad.

**Resultados:**

Se seleccionaron 17 aplicaciones, que incluían alrededor de siete habilidades de autogestión. Las aplicaciones que cumplieron con la mayoría de las habilidades de apoyo a la autogestión fueron Pathways, Branch, Pancea, Pain Navigator y Curable. Las aplicaciones Curable, Branch y MoovButh obtuvieron las puntuaciones más altas, con diez características en la escala de funcionalidad.

**Conclusión:**

Algunas aplicaciones tienen potencial para complementar el tratamiento presencial en términos de validez, aceptabilidad y utilidad clínica en el manejo del dolor. Sin embargo, barreras como la falta de asociación entre los profesionales de la salud y los pacientes, el contenido limitado basado en evidencia, el apoyo social, la relevancia cultural, el costo, el idioma, la seguridad y la privacidad pueden limitar su uso sostenido. Registro PROSPERO: CRD42022382686.

## INTRODUCTION

Pain is a complex phenomenon and demands management by healthcare providers, and can be defined as “an unpleasant sensory and emotional experience associated with actual or potential tissue damage, or described in terms of such damage”^(1)^, involving treatment, control and prevention^(2)^.

Low back pain is one of the main causes of disability in the world. When chronic, persisting for more than three months, it represents a complex and unique experience for each individual^(3)^. For the majority of people with chronic low back pain (CLBP), it is not possible to reliably identify a specific nociceptive contributor and is categorized as “nonspecific”^(4,5)^. Furthermore, it results in absenteeism, decreased labor productivity and socioeconomic impacts^(6)^, and its prevalence continues to increase. CLBP, previously seen as a symptom of an underlying condition, is now recognized in the International Classification of Diseases as a primary disease with multiple interacting factors, including biological, psychological and social aspects^(7)^.

Self-management support adapted to patients’ individual needs is recommended, incorporating techniques such as self-efficacy, self-monitoring of symptoms and Cognitive-Behavioral Therapy^(8)^. Treatment includes education, counseling, and strategies to improve patient adherence^(9)^.

The accelerated growth of health information technologies has enabled using mobile applications (apps) for tracking and self-management of pain in the context of prevention, promotion, disease control, surveillance and monitoring^(2)^. Its advantages, such as coverage of wide geographic areas, personalization based on user preferences or characteristics, and digital communication channels with messages targeted at specific groups or individuals, favor self-management and prevention both in institutional healthcare environments and in private spaces^(10)^. These actions are facilitated by the properties of mobility, portability, functionality and connectivity^(6)^.

Research using apps reveals diverse results. A study that used the International Classification of Functioning, Disability and Health (ICF) concludes that the overall quality of apps is low and offers few outcome measures to monitor effectiveness in managing low back pain^(11)^. Other research showed that none of the apps were tested on people with persistent pain or provided culturally adapted information. Features such as setting individual goals and sharing information with healthcare providers are rare^(12)^. A systematic review (SR) indicated that mobile solutions can have a positive impact on people with low back pain^(5)^. However, engagement and information need to be improved in most apps. Furthermore, it is essential that the use of tools applied to health is based on scientific evidence^(13)^. It was observed that apps have low quality and limited functionality, requiring a design centered on users’ conditions and implementation in partnership between the industry and researchers^(14)^. There are issues related to interface and security, and no pain apps suitable for clinical use have been identified^(15)^.

Self-management interventions are interdisciplinary, providing tools and strategies that support the adoption of healthier behaviors and promote collaborative care between patients and clinicians^(8)^. However, patients often demonstrate poor adherence to treatment options. It is necessary to assess the quality of these tools to validate their benefits and credibility^(14)^. Just analyzing app users is not enough to obtain all the necessary information; it is necessary to use validated scales and compare them with current best practice guides^(16)^.

Additional studies are needed to understand self-management decision making through health apps, making it a challenge for healthcare providers to identify the best applications among the many available. The literature is still limited regarding the effects of these tools focusing on self-management. Furthermore, its applicability in clinical settings remains uncertain^(17)^. Commercially available apps often lack clinical trials, criteria and standards of quality, effectiveness and evidence-based content^(11)^. Therefore, it is necessary to determine the availability, quality and resources of apps aimed at managing low back pain. In this context, a review was conducted on digital platforms with the aim of identifying and analyzing the resources and quality of self-management support of apps available in Brazil for CLBP in adults.

## METHOD

### Study Design

This is an SR carried out on digital platforms, registered in the International Prospective Register of Ongoing Systematic Reviews (PROSPERO), under registration number CRD42022382686 of December 19, 2022. The study was conducted in accordance with Preferred Reporting Items for Systematic Reviews and Meta-Analyses (PRISMA) checklist recommendations^(18)^. The methodological stages followed were: (1) research question or objective formulation; (2) protocol preparation; (3) eligibility criterion definition; (4) conducting the final search and screening of health applications; (5) data extraction; (6) assessment of quality, functionality and other relevant aspects; and (7) analysis and synthesis of results^(19)^.

### Search Strategy

The study’s guiding question was: what are the main characteristics and quality of apps available in Brazil for self-management of low back pain in adults? To structure the research question, the model represented by the acronym TECH was adopted, developed by a group of researchers who provided an overview of methodological considerations for app analysis^(19)^: Target Use: adults; Evaluation Focus: self-management skills, features and quality of apps; Connectedness: independent apps; Health Domain: low back pain.

Selection was carried out in digital stores through simple searches, using keywords in Portuguese, English and Spanish, such as *Lombalgia* (Low Back Pain, *Dolor Lumbar*), *Coluna Vertebral* (Spine*, Columna Vertebral*), *Dor nas Costas* (Back Pain, *Dolor de Espalda*), *Manejo Da Dor* (Pain Management, *Manejo del Dolor*), *Autocuidado* (Self Care, *Autocuidado*).

### Selection Criteria

Apps that incorporate at least one pain self-management strategy, such as education, exercise, psychological strategies such as Cognitive-Behavioral Therapy, monitoring and complementary therapies such as meditation, as well as promoting general physical well-being, including good sleep hygiene, were included. Furthermore, apps must be interactive, i.e., require user input to enter personal data or make choices. Apps may be available for free or for a fee, in Portuguese, English or Spanish, on platforms in Brazil, aimed at users over 18 years of age.

Apps intended exclusively for describing spinal disorders, addressing causes, symptoms, diagnosis, risk factors, etc., designed for use by healthcare providers in their daily clinical practices, such as those aimed at identifying risk factors using patient assessment instruments or aimed at spinal diagnostic tests, whose content is limited only to general information related to the spine, i.e., without any interactive component with users that offers a specific treatment plan, presenting only informational text and images with no activities to actively engage users, presenting only lists of pain management services, which were updated for more than two years, do not require additional support or ongoing technical support, and have poor functionality, were excluded. There was no restriction on the app’s release date, as long as they are compatible with the latest Android and iOS updates.

### Data Collection

Data collection took place between May and June 2023, through access to the Apple Store^®^ and Google Play^®^ virtual stores, using an individual smartphone. The selection of these operating platforms was based on their broad participation in the app market.

### Study Protocol

App selection was carried out in a single moment based on the stores’ titles and descriptions. Eligible apps were downloaded and assessed according to pre-established criteria, being analyzed for at least ten minutes or until all components were assessed. Those available on both platforms were downloaded exclusively from Google Play^®^. Both the free version and all the features available during the trial period were appreciated, including the possibility of purchasing paid versions. A search was carried out in the PubMed databases for each selected app, identifying published scientific articles and theoretical structures used in development and using the app’s name as a keyword until May 2023. Data collection was conducted independently by two evaluators.

### Study Variables

The variables included were: name; class (health and fitness, medical, educational, entertainment); developer (individual or organization); digital store (Apple Store^®^ and Google Play^®^); app description; acquisition (paid/free); characteristics (functionalities); assessment (scale from 0 to 5 points); cost and file size; number of downloads; type of pain; application purpose; app update; language; file size; health information and warnings; theoretical basis; and items from the Self-Management Support Assessment Tool (SMS-14) and Institute for Healthcare Informatics (IMS) Functionality Scoring System scale.

### Bias Risk Assessment (Quality)

SMS-14 was used, which is a 14-item personalized self-management verification scale, covering six basic self-management skills (12 items) and two functions (two items). SMS-14 was developed based on the Stanford Self-Management Support Model, Persistent Pain Guidelines, and previous reviews on self-management and pain^(8)^.

Each item is worth one point^(8)^. Skills comprise self-efficacy building through recommended pain management strategies (seven items: pain education, activity pacing, thoughts and behavioral management, exercises (biomechanical/aerobic), breathing/relaxation, meditation/mindfulness and distraction techniques), problem solving (one item: have a plan for dealing with crises), self-tailoring (one item: enables individuals to incorporate learned self-management strategies to meet their individual needs), self-monitoring (one item: thought diaries, activity self-monitoring, pain diaries, mindfulness, sleep management) and partnership between views of patient and healthcare providers (one item: through communication skills for shared decision making). Functions include providing access to social support (one item: access to a community of people living with persistent pain) and culturally tailored information (one item), which addresses cultural beliefs related to ethnicity, religion, socioeconomic status, disability, and sexual orientation^(8)^.

Functionality was assessed by the Intercontinental Medical Statistics Institute for Health Informatics Functionality Scoring System, also known as IMS Functionality Score, a validated^(20)^ app rating scale developed by the Institute for Healthcare Informatics functionality^(19)^. This instrument assesses whether or not each application meets 11 suggested features (seven items and four sub-items), assigning one point for each positive response and zero for each negative response^(19)^.

IMS seeks to identify the functions available in apps: inform (provides information in a variety of formats (text, photo and video)); instruct (provides instructions to the user (e.g., app user guides, instructions to interpret sleep charts); record (capture user-entered data); collect data (able to enter and store health data on individual phone); share data (able to transmit health data (e.g., export, upload, email sleep data)); evaluate data (able to analyze the entered health data by patient and provider, provider and administrator, or patient and caregiver); intervene (able to send alerts based on the data collected or propose behavioral intervention or changes (e.g., smart wakeup alarm based on user sleep data, anti-snoring alerts when snoring is detected)); display (graphically display user-entered data/output user-entered data (e.g., sleep trends chart)); guide (provide guidance based on user-entered information, and may further offer a diagnosis, or recommend a consultation with a physician/a course of treatment (e.g., recommendations for improving sleep based on user sleep data)); remind or alert (provide reminders to the user (e.g., bedtime notification)); and communicate (provide communication between healthcare providers, patients, consumers, caregivers and/or provide links to social networks (e.g., email or update sleep data to Facebook)^(19)^.

### Data Treatment and Analysis

Categorical variables were represented by absolute and relative frequency, whereas quantitative variables were presented by mean and standard deviation and/or median, presented in tables. The Kappa index was used to verify inter-examiner reproducibility (judge 1 and judge 2). This statistic varies in the range of [0; 1], with values closer to 1 indicating agreement between evaluators. When significant, agreement can be classified into the following ranges: poor (0–0.19); fair (0.20–0.39); moderate (0.40–0.59); substantial (0.60–0.79); and almost perfect (0.80–1.00)^(21)^. To assess the agreement between the two evaluators in SMS-14 and IMS, the mean differences between the scores (judge 1 and judge 2) were compared using t-test for related samples.

As for SMS-14 and IMS, descriptive analyzes of item responses were carried out to characterize the apps, presenting absolute, relative frequencies, means and medians. For quantitative variables, the distributions of SMS-14 and IMS items were compared between the categories of variables studied using the Kruskal-Wallis test. When significant, distribution was compared pairwise using Dunn’s post-hoc test*.* Spearman’s correlation was used to verify the degree of relationship between SMS-14 and IMS items and quantitative variables (assessment, monthly cost and file size). Correlation intensity can be classified as: weak, from 0 to 0.3; regular, from 0.4 to 0.6; strong, from 0.6 to 0.9; and very strong, from 0.9 to 1.0^(22)^. Statistical significance was established at p < 0.05. Data analysis was performed using IBM Corp. Released 2017. IBM SPSS Statistics for Windows, Version 25.0. Armonk, NY: IBM Corp.

## RESULTS

**Figure 1:**
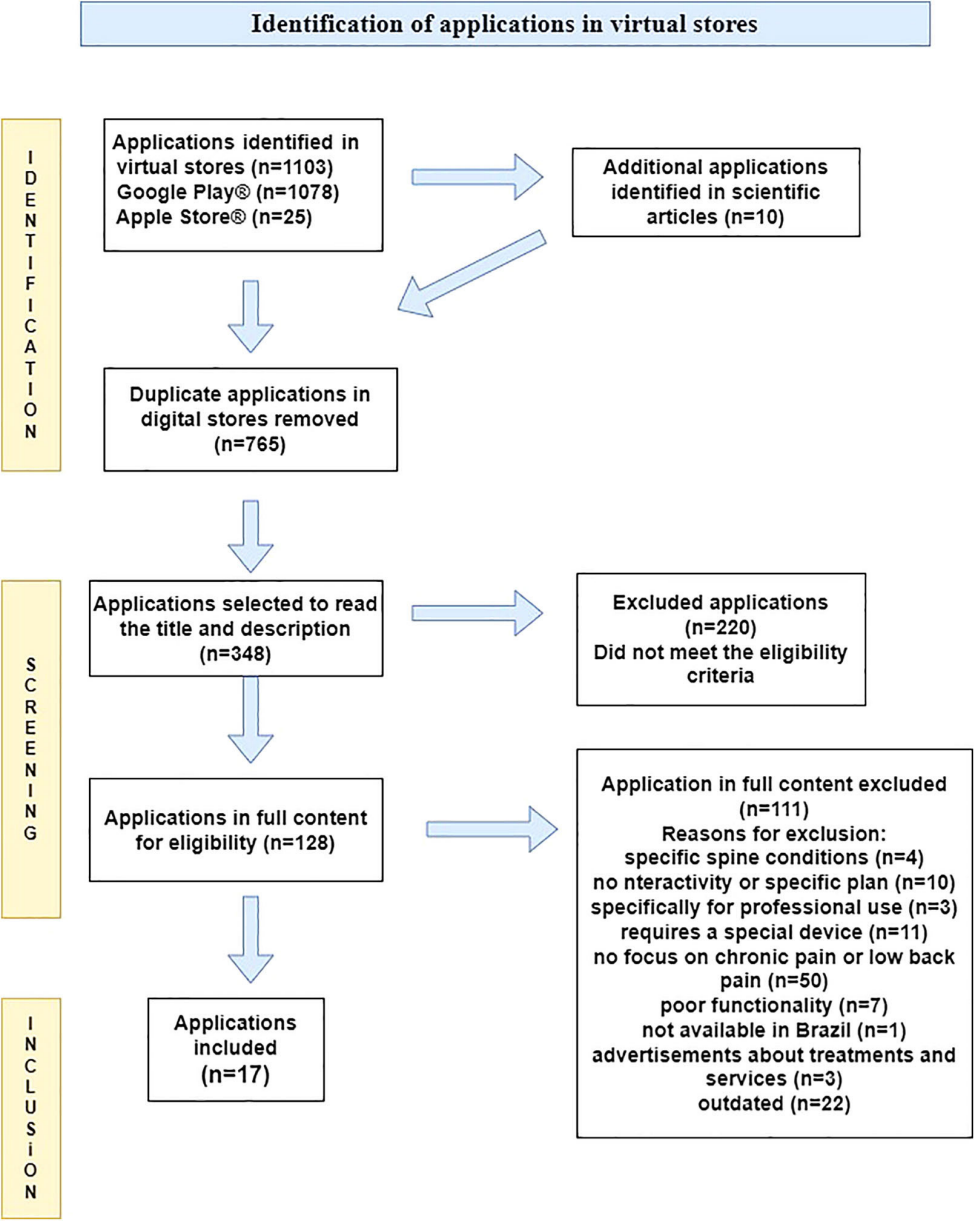
Flowchart of selected applications – Florianópolis, Santa Catarina, Brazil, 2023 (n = 17).

A total of 348 potentially relevant apps were identified during the selection process, of which 17 were considered eligible for inclusion in the review (Figure 1). Regarding agreement between evaluators in app selection, the Kappa indices of 0.828 (p < 0.001) for Google Play^®^ and 0.818 (p < 0.001) for Apple Store^®^ were higher than 0.80, indicating perfect agreement.

In relation to the area of pain, seven (41.2%) were related to back pain (low and cervical) (Back Pain Relief Exercises at, Back Pain Relief Exercises & Yo, Straight Posture – Back exercise, Perfect Posture, MoovBuddy, Vivify and Health Spine); two (11.8%) focused exclusively on low back pain (Atlas Low Back Pain and Pain Navigator); and eight (47.0%) addressed chronic pain in various areas, including the lower back (Pathways, Manage My Pain, Body Guide, *Alívio,* Curable, Branch, Pancea and Mavy). Of the 17 apps selected, two (11.7%) were found exclusively on Google Play Store^®^ (Back Pain Relief Exercises & Yo and Straight Posture – Back exercise), whereas three (17.6%) were only available on Apple Store^®^ (Atlas Low Back Pain, Health Spine and Pain Navigator).

A little more than half of the apps (52.9%) had free versions, but with advertisements and the offer of purchases to access expanded functions. Some features were unlocked by watching advertisements. Paid versions required monthly or annual subscriptions to access advanced levels of treatment, feedback and other features, such as increased difficulty and personalization of exercises, unlimited exercise plans, communication with professionals and no advertisements. Three apps (17.6%) were free (*Alívio*, Branch and Pain Navigator), and five apps (29.4%) (Pathways, Manage My Pain, Pancea, Vivify and Atlas Low Back Pain) were paid.

Access prices ranged from US$2.04 to US$12.77 per month. The number of downloads also varied, reaching from more than 1,000 to 1 million downloads. File sizes ranged from 5.72 MB to 114 MB, the largest being Branch and Vivify, respectively. The mean star rating by users was 4.4 (±0.5), and they were available on 11 (64.7%) apps. Hence, 12 (70.6%) apps presented updates in 2023, three (17.6%) in 2022 (Back Pain Relief Exercises at, Pathways and Mavy), and two (11.8%) in the second half 2021 (*Alívio* and Atlas Low Back Pain).

Goals involved improving pain, managing pain, mind-body exercises for pain relief, improving posture, reducing stress and anxiety as well as promoting health and improving well-being. The “Health and fitness” class was present in 11 (64.7%) apps (Back Pain Relief Exercises at, Back Pain Relief Exercise & Yo, Straight Posture – Back exercise, Perfect Posture, Pathways, Body Guide, Pancea, Mavy, MoovBuddy, Vivify and Health Spine); the “Medicine” class was present in five (29.4%) apps (Manage My pain, Curable, Branch, Atlas Low Back Pain and Pain Navigator); and the “Educational” class (*Alívio*) was present in one (5.9%) app. Table 1 shows the general characteristics of the apps selected in this review.

**Table 1.** General features of mobile applications – Florianópolis, Santa Catarina, Brazil, 2023 (n = 17).

Applications/version/country	Description	Developer	Language	Size	Rating
**Back Pain Relief Exercise at/1.0.116/Cyprus** 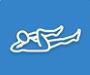	Exercises to relieve pain: back and cervical region.	Vladimir Apps	Language options	40 Mb	4.8
**Back Pain Relief Exercises & Yo/8.0/United States** 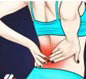	Medical research-backed mind-body-soul therapy program of Ancient Yoga, exercise and Vedic diet.	Dr Zio	Language options	21.75 Mb	4.6
**Straight Posture – Back exercise/3.4.8/Russia** 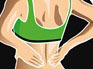	Different exercise programs for a healthy spine.	mEL Studio	Language options	25 MB	4.8
**Perfect Posture/2.7.6/United States** 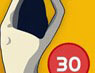	Brief exercise program to correct posture and provide a healthy spine.	Jet fitness LLC	Language options	13.64 Mb	4.7
**Pathways/2.20.9/England** 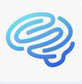	Chronic pain relief program that combines mind-body therapies.	Vortex Media Ltda	English	15.58 Mb	4.2
**Manage My Pain/4.09.2489/Canada** 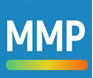	Validated digital solution for pain monitoring.	ManagingLife, Inc.	Language options (excludes Portuguese)	22 Mb	4.6
**Body Guide Pain Relief Exercise/2.8.16/Australia** 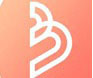	Holistic pain relief program divided into phases: relief, resolution and resilience.	Body Made Simple Pty Ltd	English	53 Mb	No rating
*Alívio* **/2.2.2 /Uruguay** 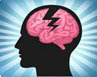	Educational game about pain and its management.	*Dor Crônica* Blog by Rosana Faria Pereira	Portuguese	40 Mb	4.4
**Curable/5.0.6/United States** 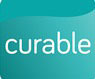	Personalized program with exercises and pain science education.	Curable Inc	English	5.72 Mb	4.3
**Branch Health: Pain Management/5.4.2/United States** 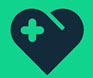	Chronic pain management through self-monitoring data, social support and rewards.	Upside Health	English	91 Mb	No rating
**Pancea – Chronic Pain Panacea/2.4.1/United States** 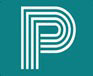	Integrated health programs to reach the root cause of individual symptoms.	Pancea, INC	English	26 Mb	No rating
**Mayv/1.3.0** 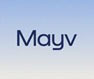	Mind-body program for personalized relief from pain, stress and other chronic symptoms.	Mayv	English	66 Mb	4.6
**MoovBuddy: your health coach/2.9.4/Turkey** 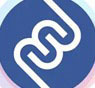	Exercise program personalized by artificial intelligence algorithm.	MoovBuddy	Language options	26 Mb	2.8
**Vivify/1.1.10//United States** 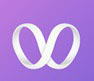	28-day program that includes pain education, meditation, exercises and guided walks in video and audio form.	Magnus Solberg	English	114 Mb	No rating
**Atlas Low Back Pain/1.0.2/United States** 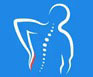	Platform with simple personalized exercises, positional corrections and psychological strategies.	Atlas Health Group Inc	English	71.5 mb	5.0
**Health Spine/1.2.5/Turkey** 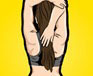	Exercise program to relieve neck and lower back pain.	Nexoft Yazilim Limited Sirketi	Language options	55.7 MB	4.7
**Pain Navigator/1.5.8/ United States** 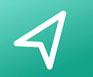	Treatment based on exercise therapy, well-being strategies, yoga, mindfulness and education.	Pain Navigator	English	27.1 mb	No rating

Some apps exclusively offered pre-established exercise programs through descriptions, videos or animated images, while others allowed programs to be customized, with a choice of exercises, series and number of repetitions. Others included educational programs, exercise and nutritional guidance. Nine (52.9%) included healthcare providers and multidisciplinary teams (Back Pain Relief Exercises & Yo, Pathways, Manage My Pain, Body Guide, Curable, Branch, Pancea, Vivify and Pain Navigator). The developers of two apps (Pathways and Curable) mentioned ongoing studies. Two apps (Pathways and Branch) involved users with pain in their development process, and only two (11.8%) apps were tested in clinical research (Manage My Pain and Pain Navigator). Manage My Pain was considered acceptable by the majority of patients in an academic pain management program during a 90-day non-randomized clinical trial. Similarly, Pain Navigator demonstrated clinical significance in CLBP treatment in a prospective pilot clinical trial.

The majority of apps (82.3%) were developed without partnerships with academic and health institutions. Manage My Pain developers have partnered with Transitional Pain Service (TPS), a multidisciplinary clinic at Toronto General Hospital. Research involving this app was conducted in collaboration with the University of Toronto and the University Health Network. Curable does not have specific articles published, but its developers have published studies on Pain Reprocessing Therapy in partnership with the Pain Psychology Center. Pain Navigator is a pain program based in the Ascension-Illinois Group – Pain Rehabilitation Ambulatory. A preliminary study involved researchers from California University of Science and Medicine, Colton, University of California San Diego and California Northstate University*.*

As for decision support interventions and meeting personalized individual needs and preferences, seven (41.1%) apps allowed users to personalize exercises (Back Pain Relief Exercises & Yo, Straight Posture – Back exercise, Perfect Posture, Body Guide, MoovBuddy, Atlas Low Back Pain and Spine Health). In two apps (11.8), personalization was carried out by the app itself, through objective assessments of movement and establishing bases for exercise programs (Pancea) and by collecting key pain markers (Atlas Low Back Pain). Four apps (23.5%) offered different exercise difficulty levels (Perfect Posture, Pathways, Health Spine and Atlas Low Back Pain), whereas eight (47.0%) allowed exercises to be performed in real time (Back Pain Relief Exercises at, Pain Relief Exercises & Yo, Straight Posture – Back exercise, Posture Perfect, Body Guide, Pancea, MoovBuddy and Spine Health).

Additionally, 11 apps (64.7%) offered statistics and activity histories (Back Pain Relief Exercises at, Back Pain Relief Exercises & Yo, Straight Posture – Back exercise, Posture Perfect, Pathways, Manage My Pain, Body Guide, Pancea, MoovBuddy, Health Spine and Pain Navigator); nine apps (52.9%) set goals (Back Pain Relief Exercises & Yo, Straight Posture – Back exercise, Posture Perfect, Pathways, Branch, Pancea, MoovBuddy, Health Spine and Pain Navigator), with Pain Navigator defining goals in the Smart format (specific, measurable, attainable, realistic and temporal); and eight apps (47.0%) used gamification systems with praise, rewards and challenges (Back Pain Relief Exercises at, Straight Posture – Back exercise, Posture Perfect, Branch, Mayv, MoovBuddy, Health Spine and Pain Navigator). In three apps (17.6%), the Body Mass Index (BMI) was calculated (Back Pain Relief Exercises & Yo, Posture Perfect and Health Spine); four apps (23.5%) included a weight chart (Back Pain Relief Exercises & Yo, Straight Posture – Back exercise, Posture Perfect and Health Spine); and four apps (23.5%) included calorie counting (Back Pain Relief Exercises & Yo, Straight Posture – Back exercise, Posture Perfect and Health Spine). Straight Posture – Back exercise offered the configuration of graphs and calculation of spinal flexibility measurements. Branch allowed participation in different communities. Regarding the interface, the apps were intuitive and easy to use, although they did not have explanations or tutorials.

With regards to self-management skills in self-efficacy building, the item “education” was present in 11 apps (64.7%) (Pathways, Body Guide, Alívio, Curable, Branch, Pancea, Mayv, MoovBuddy, Vivify, Atlas Low Back Pain and Pain Navigator) and were based on explanations about the neurophysiology of pain, the relationship between pain and psychosocial aspects and living with persistent pain. In *Alívio*, education was provided through a game. As for the origin and source of educational information, the apps claimed to be evidence-based (Pathways, Branch, Mayv, Vivity, Atlas Low Back Pain and Pain Navigator), Acceptance and Commitment Therapy (Managy My Pain), mindfulness (Pathways, Curable, Pancea, Branch, Mayv and Vivify), Pain Reprocessing Therapy (Curable), Cognitive-Behavioral Therapy (Branch), Transtheoretical Model of Change (Branch) and mindfulness (Pathways, Mayv and Pain Navigator). Psychological therapies were designed by pain management experts and involved stress management, problem solving, coping promotion, meditation, breathing, mindfulness, yoga and resilience.

In relation to health and well-being, six apps (35.3%) addressed nutritional aspects: Health Spine offered diet suggestions; Pancea provided nutrition tips; Branch presented meal plans; Back Pain Relief Exercises & Yo has made a nutrition guide available; and Pain Navigator included a food magazine. Pathways offered sleep therapies and mindful eating therapy, whereas Health Spine featured a personalized water reminder.

All apps included Terms of Use and Privacy Policy. Login and password option was available in 12 apps (70.6%) (Posture Perfect, Pathways, Manage My Pain, Body Guide, Curable, Branch, Pancea, Mayv, MoovBuddy, Vivify, Atlas Low Back Pain and Pain Navigator), while Posture Perfect offered cloud sync functionality. Branch could be offered as a commercial insurance reimbursable service. Health warnings about the risks of use and exercise were present in eight apps (47.0%) (Back Pain Relief Exercises at, Back Pain Relief Exercises & Yo, Straight Posture – Back exercise, Curable, Branch, Vivify, Atlas Low Back Pain and Pain Navigator), and in three apps (17.6%) they were presented only in Terms of Use (Straight Posture – Back exercise, Curable and Branch).

### Quality Assessment

In the *t* test for paired samples, there was no statistical difference between the differences in the means of the two judges (p > 0.05). This indicates that judges’ assessments were in agreement (SMS-14: 95%CI[-0.62; 0.97], p = 0.645 and IMS95%CI[-1.27; 0.68], p = 0.532). When discussing discordant items, consensus among evaluators was reached through discussion.

The 17 apps had a mean score of 6.9 (±2.7) and a median of 7 on SMS-14, with a minimum variation of 3 and a maximum of 11. The apps that met the majority of self-management support skills were Pathways (n = 11; 78.6%), Branch (n = 11; 78.6%), Pancea (n = 11/78.6%), Pain Navigator (n = 10; 71.4%) and Curable (n = 9; 64.3%).

Pathways and Branch met all seven items of self-efficacy building strategies. The greatest difference was the inclusion of social media components or interactive support groups for users as well as apps that addressed mind-body therapies. The most prevalent self-management skills were activity pacing, exercises (biomechanical/aerobic), breathing/relaxation, meditation/mindfulness and self-tailoring. On the other hand, skills encountered less frequently included distraction techniques, partnership between views of patient and healthcare providers for information sharing, personal support to connect with people with similar concerns, and cultural relevance with personalized information to address cultural beliefs. Apps in English had higher scores. Two free apps achieved high scores (Branch and Pain Navigator).

Based on IMS criteria, the mean number of features was 7.4 (±2.1), with a median of 8 and a minimum variation of 3 and a maximum of 10. The apps that achieved the highest scores were Curable, Branch and MoovBuddy, each with ten features. Everyone had the role of instructing. Most of the apps were intuitive, but the instructions for use proved to be important for implementing the exercises, postures and usability. Furthermore, they offered different levels of detail, and those that included videos and animations made it easier to understand.

The least frequently used features were share (n = 5; 29.4%), intervene (n = 8; 47.0%) and guide (n = 5; 29.4%). The three most common functions were instruct (n = 17; 100%), record (n = 16; 94.1%) and evaluate (n = 15; 88.2%). About the recording function, some apps allowed the recording of self-monitoring data such as pain intensity, especially those with a diary function (Manage My Pain). Other data such as weight, height and BMI were also useful and were displayed graphically. Data was presented with attractive color coding, using weekly calendar formats, graphs, and many of them recorded activity and exercise reports. Table 2 shows the descriptive analyzes of SMS-14 and IMS items.

**Table 2.** Frequencies of SMS-14^*^ and IMS^†^ items – Florianópolis, Santa Catarina, Brazil, 2023 (n = 17).

SMS-14	n	%	IMS	n	%
Pain education	10	58.8	Inform	14	82.3
Activity pacing	13	76.4	Instruct	17	100.0
Thoughts and behavioral management	7	41.1	Record	16	94.1
Exercises (biomechanical/aerobic)	13	76.4	Collect	14	82.3
Breathing/relaxation	12	70.5	Share	5	29.4
Meditation/mindfulness	12	70.5	Evaluate	15	88.2
Distraction techniques	3	17.6	Intervene	8	47.0
Self-tailoring	12	70.5	Display	11	64.7
Self-monitoring of symptoms	9	52.9	Guide	5	29.4
Goal setting and planning	10	58.8	Alert	13	76.4
Problem solving	7	41.1	Communicate	10	58.8
Partnership between views of patient and healthcare providers	4	23.5			
Social support	5	29.4			
Cultural relevance	0	0.00			

* SMS-14 = Self-Management Support Assessment Tool with 14 items

† IMS = Institute for Healthcare Informatics Functionality Score.

Table 3 shows the frequencies of SMS-14 and IMS items in each app.

**Table 3.** Frequencies of SMS-14* and IMS^†^ items in each mobile application – Florianópolis, Santa Catarina, Brazil, 2023 (n = 17).

Applications	SMS-14	IMS
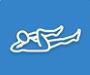	3 (21.4%)	4 (36.4%)
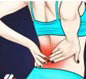	6 (42.8%)	7 (63.6%)
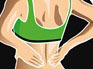	5 (35.7%)	5 (45.4%)
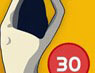	6 (42.8%)	8 (72.7)
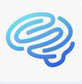	11 (78.6%)	9 (81.8)
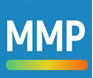	3 (21.4%)	9 (81.8)
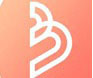	8 (57.1%)	9 (81.8)
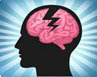	3 (21.4%)	3 (27.3)
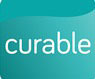	9 (64.3%)	10 (90.9%)
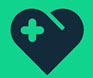	11 (78.6%)	10 (90.9%)
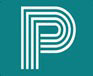	11 (78.5%)	9 (81.8%)
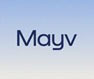	7 (50.0%)	6 (54.5%)
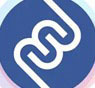	7 (50.0%)	10 (90.9%)
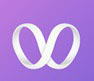	7 (50.0%)	6 (54.5%)
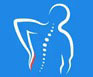	6 (42.8%)	8 (72.7%)
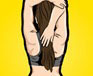	5 (35.7%)	6 (54.5%)
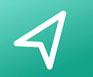	10 (71.4%)	8 (72.7%)
**Total (mean, SD^‡^)**	6.9 (±2.7)	7.5 (±2.1)

* SMS-14 = Self-Management Support Assessment Tool with 14 items

† IMS = Institute for Healthcare Informatics Functionality Score

‡ SD = standard deviation.

Comparison between the distributions of SMS-14 and IMS items between different categorical variables (class, digital store, acquisition, number of downloads, type of pain and language) using the Kruskal-Wallis test showed that there is a significant difference in the distribution between SMS-14 items between the language variable categories (p = 0.016). The English language (median = 9; mean = 8.5; SD = 2.22) differed, presenting higher scores in the distribution of language options (median = 6; mean = 5; SD = 1.67). In SMS-14, the highest scores were found in apps in English (p = 0.01). In the other comparisons, involving the two scales, no differences were found. Spearman’s correlation to verify the degree of relationship between quantitative variables (assessment, monthly cost, number of evaluative users and file size) with SMS-14 and IMS items did not show any significant correlation.

## DISCUSSION

The review identified and assessed self-care apps for low back pain in Brazil. These apps had at least three abilities and functionalities from SMS-14 and IMS. Of the apps analyzed, Pathways, Curable, Branch, Pancea and Pain Navigator obtained the highest scores on SMS-14 and scored at least five of the seven items related to the self-efficacy building skill. They had attractive interfaces, but the English language is a complication for users who do not have command of that language. These apps offer important skills for self-management of low back pain, such as mind-body therapy, social support, distractions, and evidence-based information for chronic pain relief. They also allow sharing of data with healthcare providers, questions to experts and provide virtual training to help users cope with pain. Additionally, they offer stories of new beginnings and access to communities of people experiencing chronic pain.

Some apps had specific abilities or unique tasks, which may be desirable. For instance, Manage My Pain received a low score, but offers the ability to self-monitor (pain diary) associated with positive health outcomes over time and which may contribute to reducing the psychological aspects related to chronic pain^(23)^. Apps with diary functions have the potential to improve pain management by collecting real-time data and avoiding memory bias^(2,15)^. *Alívio* provides distraction and evidence-based education about chronic pain, although scientific language may be a barrier for some users. In this regard, low scores may not necessarily reflect the quality of the apps, which require a more careful assessment.

Exercise-based apps (biomechanical/aerobic), such as Back Pain Relief Exercises at, can serve as activity pacing and be useful for specific subgroups of patients or as support associated with other more comprehensive apps. The central focus for CLBP self-management is guidance on staying active, education, and exercise. Exercise provides pain relief, functional improvement and has a positive impact on the psychological state, reducing fear and anxiety and improving quality of life^(13,24)^. These apps offer an easily accessible alternative to self-dedicated and compliant patients with back pain, as they are comparable to standard treatment^(25)^. A prospective cohort study concluded that exercise improved disability and mediated the effect of increased self-efficacy on pain relief in patients with CLBP^(26)^.

In the context of exercise, it is essential to consider compliance, user preferences, and security. Including elements such as personalized exercise programs, audio and video instructions, and reminders is key to increasing interest and engagement^(5)^. These features were identified in several apps analyzed. Furthermore, performing exercises in real time offers immediate feedback, helping to self-monitor progress and engage users^(25)^. Although some apps allow to personalize the intervention based on users’ initial profile, the effectiveness of this approach is not yet completely clear^(13)^.

Security is a concern, as apps rarely specify which exercises are suitable for autonomous or supervised practice, indications and contraindications, nor do they warn about restrictions related to users’ health. Hazards such as falls can arise without adequate supervision^(13,25,27)^. Therefore, it is crucial to investigate the quality and effectiveness of these tools to avoid health risks. Although exercises can help reduce pain, they should not replace in-person care, as individual needs vary and other strategies may be more effective in different cases, strengthening adherence^(24,28,29)^. However, exercise apps can complement the care for people with low back pain, especially in remote environments^(4,30,31)^.

Most of the apps analyzed did not have health information or alerts, which raises concerns about data privacy, security and potential risks for users. It would be important to seek advice from a healthcare provider before use^(13)^. Lack of security also makes app development more risky and compromises its quality^(32)^. Regarding data privacy, including a login and password can be a first step, but specific information about the privacy policy is equally necessary to ensure user data security^(13)^. Furthermore, governments must oversee the quality of health apps, promote evidence-based platforms, and increase public awareness about their benefits and risks^(33)^.

A little more than half of the apps provided some type of education, although not always comprehensively, considering the condition’s biological, psychological and social aspects. In Pain Navigator, education addresses stages to interrupt the vicious cycle of pain, presenting sequential modules that require completion of one to access the next. Curable offers comprehensive education on the science of pain through audio lessons through progressive learning chunks. In turn, Mavy offers classes that address psychological factors and treatment techniques. People with low back pain need accessible, structured and updated information^(34)^. SR shows that education about active management, exercise and neurophysiology of pain is effective in treating and possibly preventing low back pain^(35)^. Turning intentions into actions is essential. Education and information about the benefits of healthy behaviors are effective strategies for influencing socio-cognitive factors, such as attitudes and efficacy beliefs, leading individuals to adopt desired behaviors and promoting behavioral changes^(13)^.

Self-management requires motivation and confidence, which can be challenging for individuals with depressive symptoms or high levels of emotional stress^(3)^. It is important to increase self-efficacy through strategies, which were present in all apps, even if in a minimal way, whether through activity pacing or more comprehensively in apps with higher scores, including psychological therapies. The way one perceives and deals with pain and low self-efficacy beliefs are predictive of a worse recovery in CLBP^(36)^. Increasing self-efficacy is associated with improvements in physical function regardless of pain intensity^(36)^. In this regard, therapies such as Cognitive-Behavioral Therapy can be beneficial to reduce pain, disability and depressive symptoms and improve pain cognitive assessment^(12,36)^. Thus, individuals’ ability to control symptoms can be strengthened^(8)^.

Apps with more robust features to increase adherence and engagement have greater potential to improve patient self-management and serve as a support tool^(29–34)^. However, certain essential functionalities, such as sharing, intervening and guiding, are less common in the apps analyzed as well as partnership functions between healthcare providers and patients and social support. Some apps had the sharing function with other users exclusively for promotional purposes for the application itself. Online communities dedicated to sharing clinical experiences can contribute to a better pain prognosis, increasing self-efficacy and self-reported general health as well as reducing social isolation^(37)^. Confidentiality and privacy must be considered when incorporating these features into apps. However, it is essential to highlight that individuals with persistent pain value support and validation from their peers as essential skills for long-term self-management^(8)^.

Perfect Posture, Manage My Pain, Curable, Branch and Health Spine provide data and reports to healthcare providers. The graphical representation of pain levels over time could help professionals in the care for patients with chronic pain, allowing more efficient use of this information^(15,38)^. This would enable users to set meaningful goals, monitor their symptoms and identify, together with healthcare providers, effective strategies to improve pain, in addition to functionality^(8)^.

Personalized features, gamification, feedback and real-time self-monitoring during activities are essential to promote motivation and engagement among important subgroups^(10,38)^. For pain patients to successfully establish the rhythm of daily activities, it is necessary to plan in advance, set individualized activity goals, organize daily activities according to the condition and gradually progress activities according to progress^(4)^.

No app showed cultural relevance with culturally adapted information. Individuals with cultural and linguistic diversity who suffer from persistent pain face additional challenges due to a lack of cultural understanding and the absence of interventions adapted to their individual cultural beliefs, which makes access, acceptance and adherence to specialized pain services difficult^(8)^. It is important to ensure easy accessibility to different groups of people^(10)^.

Most of them were compatible with the Android system. To use a pain management app, system must be compatible with at least two major mobile operating systems, such as iOS and Android^(15)^. Free apps often contained ads and purchases, and some only released certain features and functionality for a fee. Therefore, the cost of downloading the app is one of the factors to be considered, as some require a one-time payment to be downloaded, whereas others adopt a subscription model^(25)^ and, therefore, may not be accessible to the entire population. Cost-effectiveness studies are needed to justify the change in the payment structure of pain management apps and to justify the adoption of an empirically supported app by private and public healthcare systems^(38)^.

There was a certain limitation in content relevance in relation to app effectiveness and quality, since only two of them (Manage My Pain and Pain Navigator) have been evaluated in people with pain and few indicated that the source of information was evidence-based. Users themselves suggest that evidence-based information and medical advice are important criteria^(37)^. The feasibility study concluded that the Pain Navigator platform can be used to treat low back pain, as it presents biopsychosocial effect measures, modalities focused on users’ needs and is clinically important. The focus is not just on pain levels, but also on functionality^(28)^. Manage My Pain was considered acceptable by the majority of patients in an academic pain management program. User registration and retention rates were favorable compared to other apps^(39)^.

Most pain management apps are untested and have low quality and limited functionality, making it difficult to assess their effectiveness relative to conventional treatments. There is an urgent need for partnership between industry and researchers to develop apps that are better adapted to users’ needs and the context of health conditions, including consideration of culturally adapted information^(8,14)^. The lack of scientific basis, healthcare providers’ involvement and adequate regulation are critical concerns^(2,11,39,40)^. Collaboration between healthcare providers and developers can improve the quality and adoption of these apps, which must be designed with usability and interface appropriate to the target audience^(15,25)^. The need for development by scientific institutions stands out^(41)^. Although validated clinical content is essential, if the application is not intuitive and easy to use, it leads to errors and increases the time to perform tasks, leading to less willingness to use it^(15)^. However, in health tools, the scientific basis, security and privacy must always prevail as priority aspects in relation to usability^(41)^.

There was no relationship between file size, star rating, number of user reviewers, and SMS-14 and IMS scores. This suggests that user ratings alone do not reflect app quality, as the apps with the highest scores were not necessarily the best rated by users. Assessments carried out by users can be highly subjective and lack scientific value, which can make it difficult for other users to decide when choosing apps^(11)^. This subjectivity may involve factors such as individual expectations, personal experiences, emotional factors and the diversity among users.

An app, if introduced appropriately and as a complement to care, can be an empowering tool for self-management^(27)^. However, for effective integration into clinical practice, it is crucial to develop them with a focus on clinical implementation. One challenge is inconsistency in the use of data from apps by healthcare providers, who rarely integrate them into electronic medical records. It is vital to involve users (healthcare providers, patients and families) in development and evaluation to meet needs and ensure clinical applicability^(38)^.

Defining the ideal features for a health app represents a significant challenge. The tool must encourage behavioral changes and maintain a high level of engagement, taking into account functionality, usability and design. The quality of information is crucial for security and effectiveness, requiring adequate testing. Furthermore, it is essential to adhere to current regulations to ensure user privacy^(13)^.

The ideal app would incorporate features such as education to help users better understand their condition, exercises with video or audio tutorials accompanied by health alerts, self-monitoring of symptoms so users can record their daily symptoms, activities and pain levels and thus identify patterns and triggers. It should also allow setting goals, sharing information with healthcare providers and other users, including features to increase engagement, such as reminders and notifications. Additionally, gamification, relaxation and meditation techniques could be integrated to help reduce stress and muscle tension, which can aggravate low back pain. The interface must be pleasant and the application must be interactive. It is essential that it is evidence-based, easy to use and customizable to meet users’ needs. Therefore, more studies are needed to evaluate the effectiveness of existing and new tools, providing a comprehensive analysis that offers relevant information to users for a more appropriate choice^(13)^.

### Study Limitations

The study assessed apps available only on digital platforms in Brazil. Thus, there is no knowledge about the quality of other apps available on platforms in other countries. Due to the constant updating of the app market, the results presented may not reflect the current situation with the latest updates or new apps released after the assessment period. However, the objective was to highlight important skills present in the apps to guide users in self-management. Some paid apps, which require monthly or annual subscriptions, limit access to all features. Even so, some were purchased or tested during the free usage period based on description, screenshots, videos, and user reviews. Another limitation is that the instruments used in assessment have not yet been validated in Brazil. SMS-14 employed a “yes” or “no” score without assessing the quality or depth of the topic covered. Furthermore, apps that did not use the word “pain” in their title or description may not have been captured, although additional searches were carried out in databases. Finally, this study does not make recommendations about which app to use, but it does offer insights into the quality and characteristics of the apps assessed.

## CONCLUSION

The study analyzed apps aimed at low back pain self-management and identified that some of them have the potential to complement in-person treatment in terms of validity, acceptability and clinical usefulness. However, most apps do not present robust and scientifically proven self-care strategies and fail to include important features, which creates uncertainty regarding their benefits. Few apps partner with healthcare providers or offer social support, and none address cultural or diversity issues. Other barriers, such as cost, language, security, and privacy, may limit its sustained use. It is essential to consider them as a complement to care, not as a substitute, and to assess them through a formal scientific assessment. Therefore, it is suggested that studies be developed to assess existing apps or those that may be developed, as well as collaboration between developers, healthcare providers and users with pain, providing relevant information for the appropriate choice of an app with clinical utility.
